# Supplementary material for: Identification of photoperiod-induced specific miRNAs in the adrenal glands of Sunite sheep (Ovis aries)
Source: Front Vet Sci. 2022 Jul 22;9:888207. doi: 10.3389/fvets.2022.888207 (PMC9354845; doi:10.3389/fvets.2022.888207)
Supplement: Supplementary file 1 [file Data_Sheet_1.ZIP › Supplementary file/miRDeep2 file.zip!/miRDeep2 file/Know_Structure/SP42A1/oar-mir-382.pdf]

miRBase precursor : oar-mir-382
Total read count : 3010
oar-miR-382-5p read count : 1186
oar-miR-382-3p read count : 1821
remaining reads : 3

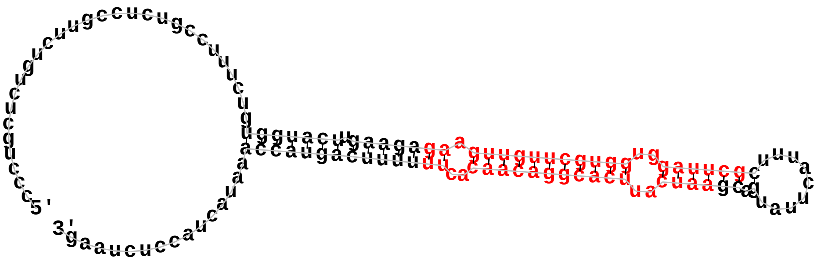

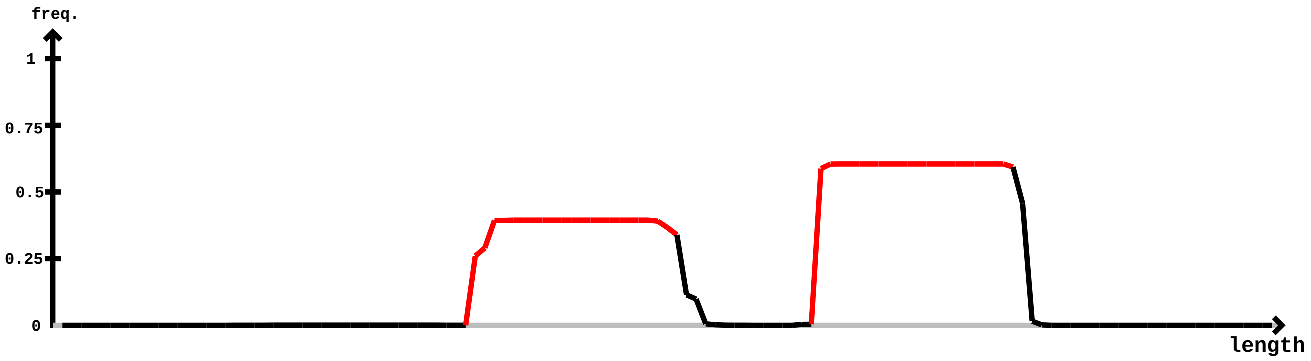

oar-miR-382-3p

oar-miR-382-5p

```
5'- cccugcucugucuugccucugccuuucugugguacuugaagagaaguuguucgugguggauucgcuuuacuuaugacgaaucauucacggacaacacuuuuuucaguaccaaauacuaccucuaag   -3'    exp
    ....((........)).............(((((((.(((((((.(((((((((((..(((((((.........).))))))..)))))))))))..))))))))))))))...............    reads    mm
    .................ucugccuuucugugguacuuga.......................................................................................    1        0
    .....................ccuuucugugguacuugaaga....................................................................................    1        0
    ..........................................gaaguuguucgugguggaG.................................................................    1        1
    ..........................................gaaguuguucgugguggau.................................................................    10       0
    ..........................................gaaguuguucgugguggauu................................................................    69       0
    ..........................................gaaguuguucgugguggauuc...............................................................    76       0
    ..........................................gaaguuguucgugguggauucg..............................................................    593      0
    ..........................................gaaguuguucgugguggauucgc.............................................................    32       0
    ..........................................gaaguuguucgugguggauucgcu............................................................    1        0
    ...........................................aaguuguucgugguggauu................................................................    3        0
    ...........................................aaguuguucgugguggauuc...............................................................    6        0
    ...........................................aaguuguucgugguggauucg..............................................................    81       0
    ...........................................aaguuguucgugguggauucgc.............................................................    2        0
    ............................................aguuguucgugguggauuc...............................................................    1        0
    ............................................aguuguucgugguggauucg..............................................................    3        0
    ............................................aguuguucgugguggauucgc.............................................................    14       0
    ............................................aguuguucgugguggauucgcu............................................................    278      0
    ............................................aguuguucgugguggauucgcuu...........................................................    11       0
    ............................................aguuguucgugguggauucgcuuu..........................................................    2        0
    ............................................aguuguucgugguggauucgcuuua.........................................................    1        0
    ..............................................uuguucgugguggauucgcuuu..........................................................    2        0
    ..............................................uuguucgugguggauucgcuuuacu.......................................................    1        0
    ............................................................................cgaaucauucacggacaacacu............................    3        0
    ............................................................................cgaaucauucacggacaacacuu...........................    7        0
    .............................................................................gaaucauucacggacaacacuu...........................    1        0
    .............................................................................gaaucauucacggacaacacuuu..........................    1        0
    ..............................................................................aaucauucacggacaacacu............................    31       0
    ..............................................................................aaucauucacggacaacacuu...........................    402      0
    ..............................................................................aaucauucacggacaacacuuu..........................    1291     0
    ..............................................................................aaucauucacggacaacacuuuu.........................    30       0
    ..............................................................................aaucauucacggacaacacuuuuu........................    3        0
    ...............................................................................aucauucacggacaacacuu...........................    1        0
    ...............................................................................aucauucacggacaacacuuu..........................    37       0
    ...............................................................................aucauucacggacaacacuuuu.........................    11       0
```

```
                                                                              oar-miR-382-3p
                                    oar-miR-382-5p
cccugcucugucuugccucugccuuucugugguacuugaagagaaguuguucgugguggauucgcuuuacuuaugacgaaucauucacggacaacacuuuuuucaguaccaaauacuaccucuaag

..............................................................................aucauucacggacaacacuuuuu........................    2    0
...............................................................................ucauucacggacaacacuuuu.........................    1    0
```
